# Neighborhood Safety and Major Depressive Disorder in a National Sample of Black Youth; Gender by Ethnic Differences

**DOI:** 10.3390/children4020014

**Published:** 2017-02-23

**Authors:** Shervin Assari, Cleopatra Howard Caldwell

**Affiliations:** 1 Department of Psychiatry, School of Medicine, University of Michigan, 4250 Plymouth Rd., Ann Arbor, MI 48109, USA; 2 Center for Research on Ethnicity, Culture and Health, School of Public Health, University of Michigan, 1415 Washington Heights, Ann Arbor, MI 48109, USA; cleoc@umich.edu; 3 Department of Health Behavior and Health Education, School of Public Health, University of Michigan, 1415 Washington Heights, Ann Arbor, MI 48109, USA

**Keywords:** neighborhood, depression, Blacks, African Americans, gender, ethnicity

## Abstract

Adolescence is a developmental period marked by increased stress, especially among Black youth. In addition to stress related to their developmental transition, social factors such as a perceived unsafe neighborhood impose additional risks. We examined gender and ethnic differences in the association between perceived neighborhood safety and major depressive disorder (MDD) among a national sample of Black youth. We used data from the National Survey of American Life - Adolescents (NSAL-A), 2003–2004. In total, 1170 Black adolescents entered the study. This number was composed of 810 African American and 360 Caribbean Black youth (age 13 to 17). Demographic factors, perceived neighborhood safety, and MDD (Composite International Diagnostic Interview, CIDI) were measured. Logistic regressions were used to test the association between neighborhood safety and MDD in the pooled sample, as well as based on ethnicity by gender groups. In the pooled sample of Black youth, those who perceived their neighborhoods to be unsafe were at higher risk of MDD (Odds Ratio [OR] = 1.25; 95% Confidence Interval [CI] = 1.02-1.51). The perception that one’s neighborhood is unsafe was associated with a higher risk of MDD among African American males (OR=1.41; 95% CI = 1.03–1.93) but not African American females or Caribbean Black males and females. In conclusion, perceived neighborhood safety is not a universal psychological determinant of MDD across ethnic by gender groups of Black youth; however, policies and programs that enhance the sense of neighborhood safety may prevent MDD in male African American youth.

## 1. Introduction

Major depressive disorder (MDD) is a pressing public health issue [1]. As the most common psychiatric disorder, MDD is the leading cause of disability and a significant contributor to the burden of disease both in the U.S. and globally [2]. About 10% of the population in the United States meets the Diagnostic and Statistical Manual of Mental Disorders, Fourth Edition (DSM-IV) criteria for MDD; however, its prevalence differs by race, ethnicity, gender, and age group [3,4,5]. Although MDD may be less common among Blacks [4,5,6], depression tends to be more chronic, severe, and disabling in Blacks [4,7]. As a result, regardless of the presence of clinical MDD, Blacks have more severe depressive symptoms than Whites [8,9]. 

Adolescence is a key stage of a child’s life with major implications for human development [10,11]. This is also true for Black youth who are exposed to a wide range of additional economic and social risk factors (e.g., poverty, poor school quality, poor neighborhood, etc.) that can contribute to behavioral and emotional problems such as MDD [12,13,14,15]. From a wide range of social risk factors, recent research has documented the unique role of neighborhood disorder on physical and mental health of Blacks, particularly, Black youth [12,16,17]. Most of this research, however, has measured depressive symptoms rather than MDD (i.e., the gold standard outcome) [18,19,20]. In addition, most of this research has used a local sample [18,20,21] providing non-generalizable findings. Finally, we still do not now know if ethnicity and gender alter psychosocial determinants of mental health among Black youth. Thus, there is a need for studying the role living in stressful conditions such as unsafe neighborhood has on the risk of MDD among an ethnically diverse nationally representative sample of Black youth [22]. 

It is during the adolescence that youth strive to achieve independence [23,24] and get more involved with a social life [25], and Black youth are not exceptions to this rule [26]. In addition to the age related normative developmental challenges that are universal across all ethnic groups, Black youth—who disproportionately live in economically and socially disadvantaged urban contexts—experience additional risk factors that impose them to specific stressors that may increase their risk of MDD [18,20,26]. Although Black youth perceive stressors in other life domains (daily life, finances, and racial discrimination) as well, neighborhood stress due to unsafe environments and the fear of violence are a major risk factor for depression and depressive symptoms among male and female Black youth [12,16,18]. 

For Black youth who live in the inner cities, neighborhood fear is a significant risk factor for depression [20]. Both exposure to violence [27,28,29,30,31,32,33,34] and also fear of it [12,16,20] deteriorates mental and physical well-being of Black youth. Black youth who live in low-income neighborhoods are more likely to witness shootings, stabbings, and killings in their neighborhoods compared to their White and middle to upper income peers [26,35]. Among Black youth, males are more frequently victims of neighborhood violence [26,36,37]. 

Even in the absence of direct exposure to violence, living in unsafe neighborhoods endangers the youth development, as fear of violence in the neighborhood is a risk factor that increases risk of undesired health outcomes [38,39,40]. Studies among adolescents [38] and adults [40] living in economically and socially disadvantaged neighborhoods suggest that perception of neighborhood disorder predicts a wide range of poor health outcomes including depression [19]. Blacks and particularly African Americans are more likely to live in low-income communities where structural inequities increase the propensity for social disorder, crime, and violence, which all place them at an increased vulnerability for the onset of MDD or depressive symptomology [40,41]. 

Fear of violence has unique implications for the mental health and well-being of African American youth during the transition to adulthood [42,43]. Developmentally, youth are expected to establish friendship bonds and focus on education which is important for their future success. In addition to the reality of limited resources and constrained opportunities [43,44], the perception of a neighborhood as unsafe and having fear of violence may increase hopelessness and decrease future orientation among Black youth [45]. In unsafe neighborhoods, survival becomes the number one priority [35,46]. 

Informed by a socio-ecological model of development [47] as well as intersectionality framework [48,49,50], the present study investigated the association between neighborhood safety and MDD in a nationally representative ethnically diverse community sample of Black youth [51,52,53]. In addition, we explored gender, ethnicity, and gender by ethnic differences in the association between neighborhood safety and MDD among Black youth. 

## 2. Materials and Methods

For this cross-sectional study, we used data from the National Survey of American Life - Adolescents (NSAL-A), 2003–2004 [54,55], the largest and the most updated mental health survey of Black youth in the U.S. history [51,52,53]. The study received funding from the National Institute of Mental Health (NIMH), as a part of the Collaborative Psychiatric Epidemiology Surveys (CPES) series.

### 2.1. Ethics

The NSAL (including NSAL-A component) was approved by the Institute Review Board of the University of Michigan (UM), Ann Arbor (B03-00004038-R1). Informed consent was obtained from youths’ legal guardians. Assent was obtained from the youth themselves. Participants received financial compensation for their time ($50). Parental informed consent and assent were obtained from all adolescent participants included in the study. All procedures performed in studies involving human participants were in accordance with the ethical standards of the institutional and/or national research committee and with the 1964 Helsinki declaration and its later amendments or comparable ethical standards.

### 2.2. Participants and Sampling

The NSAL-A included 1170 Black adolescents. This number was composed of 810 African American and 360 Caribbean Black youth between 13 and 17 years who were residing in the United States at the time of study. More details on the NSAL sampling are available elsewhere [52,53]. While African American youth were residents of either large cities or other urban and rural areas, Caribbean Black youth were limited to the large cities. The NSAL used a national household probability sample of Blacks. All African American and Caribbean Black households participating in the NSAL-Adult survey were screened for eligible adolescents who lived in the household. Adolescents were then randomly selected from the provided list. When more than one eligible adolescent was living in the household, two adolescents were selected based on the gender of the first selected adolescent. As a result, the NSAL-A sample was non-independent. As a result, the data were weighted to adjust for non-independence in selection probabilities within the households, as well as non-response rates at the households and individual levels. The weighted data were then post-stratified to represent the national estimates based on gender and age among African American and Caribbean Black youth [54,55].

### 2.3. Interview

From all the interviews, 82% were conducted face to face, in their homes, and the remaining 18% were conducted either entirely or partially by telephone. To conduct the face-to-face in-person interviews, computer-assisted personal interviews (CAPIs) were used to enhance quality of the data. CAPI, an interviewing technique in which the respondent uses a computer to answer the questions, is the preferred method of interview when the questionnaire is long and complex. All the interviews were performed in English, and each interview lasted on average 100 minutes. The overall response rate was 80.6% (80.4% for African Americans and 83.5% for Caribbean Blacks).

### 2.4. Measures

The study included demographic factors including age, gender, and ethnicity. Clinical depression (MDD) was measured using World Mental Health *Composite International Diagnostic Interview* (CIDI) [56,57], while neighborhood safety was measured using self-reported data.

#### 2.4.1. Ethnicity 

Ethnicity was measured according to the ethnic group of the parents who were living in the same household. Parents self-identified as African American if they were Black and did not have ancestral ties to any Caribbean countries. Parents identified as Caribbean Black if they were Black and they were from any of the following Caribbean countries: (1) Cuba, (2) Dominican Republic, (3) Haiti, (4) The Bahamas, (5) Jamaica, (6) Trinidad and Tobago, (7) Dominica, (8) Saint Lucia, (9) Antigua and Barbuda, (10) Barbados, (11) Saint Vincent and the Grenadines, (12) Grenada, or (13) Saint Kitts and Nevis.

#### 2.4.2. Depression 

Lifetime and 12-month MDD was measured using a modified version of the CIDI. A fully structured diagnostic interview schedule, the CIDI evaluates a wide range of Diagnostic and Statistical Manual, 4th Edition (DSM-IV) mental disorders including but not limited to MDD. Originally developed for the World Mental Health project initiated in 2000 [56], the CIDI is developed for use by trained lay interviewers to generate diagnoses of lifetime and recent DSM-IV-TR/ International Classification of Diseases -10 (ICD-10) disorders [57]. Clinical reappraisal studies have documented acceptable psychometric properties such as good concordance of CIDI diagnoses with blinded clinical diagnoses [56,57,58,59,60]. This measure provides valid findings for Blacks and their ethnic groups [61,62,63,64,65,66]. This measure also has acceptable function in youth [67,68,69,70,71,72,73,74,75]. 

#### 2.4.3. Neighborhood Safety 

A short survey was used to measure perceived neighborhood safety. The stem question was “How true is each of the following statements about your neighborhood?” Items were: (1) “I feel safe being out alone in my neighborhood during the day”; and (2) “I feel safe being out alone in my neighborhood at night” [12,16,17,76,77]. Item responses included very true, somewhat true, not very true, and not at all true. We calculated a total score, ranging from two to eight, with a higher score indicative of unsafe neighborhood. Correlation between the two items was strong (r = 0.53). 

### 2.5. Statistical Analysis

Stata 13.0 (Stata Corp., College Station, TX, USA) was used to account for the survey design of the NASL-A. We used Taylor Series approximation technique to recalculate all the standard errors (SEs) that reflect design-based weights. Proportions reported in this study are all weighted and are representative of the U.S. population. While in the NSAL-A, the Caribbean Black sample is more clustered than the African American sample, SEs are systematically larger for Caribbean Blacks than African Americans. As a result, results are more conservative in Caribbean Blacks than African Americans. 

We used survey logistic regression models to conduct multivariable analysis. In all our models, lifetime MDD was the main outcomes, perceived unsafe neighborhood was the predictor, and age, gender, and ethnicity were the control variables. Ethnicity and gender were also considered as the moderators. In the first step, the association of interest was tested in the pooled sample, controlling for the main effects of gender and ethnicity. In the next step, we ran models specific to ethnicity, gender, and their intersections. Adjusted odds ratio (OR), SEs, 95% confidence interval (CI), and *p*-values were reported. *p*-Values less than 0.05 were considered statistically significant.

## 3. Results

Table 1 provides descriptive statistics for demographic factors, neighborhood safety, and MDD in the pooled sample. Mean age of the participants was 15 years old. Participants were almost equally composed of male and females. Table 1 also shows descriptive statistics for demographic factors, neighborhood safety, and MDD based on ethnicity and gender. Age was higher in Caribbean Blacks than African Americans, and more females held the perception of their neighborhood being unsafe, compared to males.

As Table 2 suggests, perception of neighborhood as unsafe was associated with higher risk of MDD in the pooled sample (Odds Ratio (OR) = 1.25, 95% Confidence Interval (CI) = 1.02–1.51). Age was also associated with risk of MDD (OR =1.42, 95% CI = 1.15–1.75); however, ethnicity and gender did not have main effects on MDD risk.

Table 3 shows summary of four logistic regressions based on ethnicity and gender. Neighborhood safety was associated with risk of MDD in African Americans (OR = 1.24, 95% CI = 1.01–1.53) and males (OR = 1.40, 95% CI = 1.03–1.90) but not Caribbean Blacks or females.

Table 4 summarizes four logistic regressions across ethnic by gender groups. Perception of neighborhood as unsafe was associated with an increased risk of MDD among male African American (OR = 1.41, 95% CI = 1.03–1.93) but not female African American and male and female Caribbean Black youth. 

## 4. Discussion

To summarize the findings, in the pooled sample of Black youth, those who perceived their neighborhood as unsafe were at a higher risk of MDD, net of covariates. Perception of neighborhood as unsafe was associated with higher risk of MDD among African American males but not African American females or Caribbean Black males and females. These results offer new insights for policy makers, public health experts, and clinicians who wish to promote the mental health of African American males. These findings can be used to design and implement public policy, public health programs, and clinical protocols in order to prevent MDD in African American males. 

Although neighborhood is a major social determinant of mental health [78,79,80,81,82,83], a growing body of evidence has documented heterogeneity of the neighborhood-health link based on race, ethnicity, and gender [12,17,20]. Using a local sample of Black youth, a recent longitudinal study showed similar results. The study showed that an increase in perceived neighborhood fear predicts an increase in depressive symptoms among male but not female African American youth [20]. 

Our findings are in line with earlier studies examining the relationship between perceived threat and fear from neighborhood violence and crime and disorder and depression [19,20,38,40]. A sense of safety in the neighborhood is an important pre-requisite for maintaining well-being among Black youth who are more likely to live in dangerous environments and perceive fear of crime and violence. This is particularly the case for male African American youth who are at the highest risk for exposure to violence [20,26,35]. 

Goldman-Mellor and colleagues used data from 4464 adolescent respondents from the California Health Interview Survey (CHIS 2011–2014) and found that adolescents who perceived their neighborhood to be unsafe were at a higher risk of serious psychological distress (OR = 2.4). Living in areas with higher levels of violent crime, however, did not predict mental distress. These authors showed that adolescents’ perception of neighborhood violence, rather than objective levels of neighborhood crime, is a risk factor for poor mental health [84]. Curry et al. investigated how neighborhoods with high levels of violent crime deteriorate mental health of residents through risk of experiencing violence. Using data from current and former drug users (*n* = 786) nested in 270 block groups within Baltimore, Maryland, they examined the relationships among block-group level crime, perceived neighborhood disorder, violence experienced in the neighborhood, and depression. Using path analysis, the study showed that neighborhood violence increases risk of psychological distress through perceptions of neighborhood disorder. They concluded that community and structural level interventions are needed to decrease neighborhood crime as well as improve residents’ sense of neighborhood safety [85]. We also found that perceiving neighborhood as unsafe is associated with an increased risk of MDD. 

Gender differences in the effects of stressors such as neighborhood and social disorder on MDD may be due to a higher threshold of perceived risk among males [86] or role of gender roles [87] and masculinity ideologies [88] that all have major implications for the effect of stress on depression [20,89]. Higher thresholds of perceived risk among males suggest that it may be more severe when reported [86]. Thus, when a male reports a risk, it may have more consequences for his health. Similar trends have been reported for self-rated health (SRH), as poor SRH better reflects risk of death among males than females [13]. Another line of research that has shown similar findings is the link between stress and depression. Although men generally report lower levels of stress, when they report it, it comes with a higher vulnerability than females [90]. Similarly, discrimination has a stronger effect on mental health of men than women [14,15,91,92,93]. 

Males are socialized to look tough and fearless, and deny any vulnerability in the presence of stress, trauma, and social disorder [94]. This masculine mask increases the individual vulnerability to emotional problems when stress happens [88]. Male African American youth are not exempt from this rule: neighborhood related stress puts them at risk of depression [18,20]. Adherence to masculine norms is a major barrier for male African American youth to reach out and seek emotional support, when needed [95]. Living under chronic neighborhood stress, with a masculine mask, may jeopardize their mental well-being early in life. If left undiagnosed and untreated, MDD in adolescence increases risk of emotional, behavioral, and social problems across a wide range of domains later in life [96]. This is particularly important given low access and trust and high stigma that delays health care utilization for psychiatric disorders in African American males [97]. 

We found that the neighborhood–MDD link differs by the intersection of gender and ethnicity. Groups based on gender, race, ethnicity, and their intersections have specific paths to their well-being and health [98,99,100,101,102,103,104,105,106,107,108,109]. This is also known as differential vulnerability or differential effect hypothesis, suggesting that pathways and mechanisms that play a role in maintaining health and disease are specific to population subgroups that live in different contexts, and have different values, cultures, life histories, resources, etc. [110,111].

African American males experience a unique set of social and economic challenges and adversities in their life, starting from childhood and adolescence, continuing in their adulthood, and extending until older age. Some of these unique challenges include low socioeconomic status, education in low resource schools, police brutality, unjust opportunities for employment, unemployment, differential pay, discrimination, and racism [18,112]. These structural, political, and historical inequalities affect African American youths’ lives, particularly males [113,114]. Many of these high-level structural determinants are stressful and contribute to health problems (e.g., MDD) for African American males across their life span [18,115]. 

Investments to prevent neighborhood crime and violence are essential if we wish to protect African American males against MDD. This finding has major public health implications as Blacks particularly African American males have very low rates for utilizing formal mental health services [116], and interventions that try to increase their professional health care have not been very successful [117]. In a nationally representative community sample of Blacks, most (55%) of the African Americans who had MDD had not received any treatment for depression during their lifetime [4]. Williams et al. showed that more African Americans with lifetime MDD have recent diagnosis of MDD, which was indicative of higher chronicity of MDD for Blacks [4]. In another study, among those who had MDD, African Americans suffered higher burden of depressive symptoms than Whites, a pattern which is attributed to higher chronicity, more severity, and poor health care use [9]. African Americans less frequently accept medications and prefer non-pharmacological treatments of depression such as counseling [118,119,120,121]. 

Policies and programs that boost the sense of neighborhood safety of residents may have implications for mental health promotion of male African American youth. More community mental health practitioners are needed to screen for MDD among youth in the neighborhoods with poor social elements that may trigger risk of MDD among African American males. It should be noted that actual levels of crime may not predict depression risk [76,84]. Our findings advocate for the promotion of a sense of safety, which is different from crime rates. 

Clinical practice that focuses on screening, diagnosis, treatment, as well as prevention of MDD in Black youth should be aware of the within-Black heterogeneity in the social determinants of MDD. African American males who live in unsafe neighborhoods are particularly at a high risk of MDD and need to be screened. 

The study sheds additional light about how and when social and environmental factors contribute to development of MDD among Black youth, and how diversity of this group alters their vulnerability to the same risk factor. This finding offers higher level interventions at the state levels that promote safety as a strategy to promote male African American youth in general, and prevent MDD in particular. 

Our study had at least five major limitations. First major limitation is the cross-sectional design of the study, which precludes a causative interpretation of our findings. Thus, we cannot rule out “selection bias,” i.e., youth with “poor” health may gravitate toward “poor” neighborhoods. Reverse causation is also a possibility, as poor health may operate as a barrier for upward social mobility and moving out of a bad neighborhood. Second, the study is subject to measurement bias and misclassification of neighborhood safety, as we did not use a previously validated measure for neighborhood safety. In our study, the main predictor variable was based on only two survey items, thus the results should be interpreted with caution. Our measure was purely subjective, and future research should also use objective variables (neighborhood crime, poverty, resources, ethnic composition, etc.). Third limitation was that our study was limited to individual level data. Prior research has shown that violence at the neighborhood level reduce perception of safety at an individual level [81,122]. More research is needed to better understand how groups differ in the mediational path from neighborhood level factors that follow a geographic distribution to neighborhood fear to mental distress. There is also a need to study how place alters these links, as an adolescent living in an inner-city ghetto may have a different threshold for perception of neighborhood risk and fear compared to another youth living in a low crime suburb. Forth, current study did not include socioeconomic factors such as family income or family structure. We also did not have data on the duration of residence in the neighborhood. Finally, the sample size was not balanced in our gender by ethnicity groups, and standard errors are larger for Caribbean Black youth, which has implications for interpretation of the findings across groups with different sample size. Although the association did not reach significance level, we found a trend toward an association between perceived safety and MDD among Caribbean Black youth. Although the *p*-value was not statistically significant, the magnitude of the association is virtually identical to that among African Americans. Thus, the results should be interpreted with caution.

Despite above limitations, the nationally representative sample and large sample size of Black youth were major strengths of this study. Thus, the results are generalizable to all Black youth residing in the United States. However, there is still a need to future longitudinal studies that may establish a causal link between the exposure and the outcome. 

To conclude, there is a link between perceived neighborhood safety and depression among Black youth, however, this association is not uniform across all their sub-groups. Perception of neighborhood as unsafe is associated with an increased risk of MDD in African American males but not African American females or Caribbean Black males or females. 

## Figures and Tables

**Table 1:** Descriptive statistics among Black youth.

	All		African Americans		Caribbean Blacks		Males		Females	
	Mean	95% CI	Mean	95% CI	Mean	95% CI	Mean	95% CI	Mean	95% CI
Age	15.01	14.89–15.14	15.00 ^a^	14.86–15.13	15.24 ^a^	15.11–15.37	15.04	14.89–15.18	14.99	14.81–15.17
Perception of neighborhood as unsafe	3.34	3.21–3.47	3.32	3.19–3.45	3.58	3.22–3.94	2.99 ^b^	2.84–3.14	3.68 ^b^	3.50–3.86
	%	95% CI	%	95% CI	%	95% CI	%	95% CI	%	95% CI
Gender										
Male	50.02	46.49–53.54	50.39	46.57–54.20	44.78	39.98–49.68	–	–	–	–
Female	49.98	46.46–53.51	49.61	45.80–53.43	55.22	50.32–60.02	–	–	–	–
Ethnicity										
African American	93.37	91.89–94.60	–	–	–	–	94.07	92.69–95.20	92.68	90.64–94.31
Caribbean Black	6.63	5.40–8.11	–	–	–	–	5.93	4.80–7.31	7.32	5.69–9.36
Major Depressive Disorder										
No	93.72	91.89–95.16	93.75	91.75–95.29	93.37	90.80–95.25	94.09	91.58–95.89	93.35	90.31–95.48
Yes	6.28	4.84–8.11	6.25	4.71–8.25	6.63	4.75–9.20	5.91	4.11–8.42	6.65	4.52–9.69

^a^ p < 0.05 for comparison of African Americans and Caribbean Blacks; ^b^ p < 0.05 for comparison of males and females; CI: Confidence Interval.

**Table 2:** Summary of logistic regression in the pooled sample of Black youth.

	OR	SE	95% CI	*p*
Perception of neighborhood as unsafe	1.25	0.12	1.02–1.51	0.029
Age	1.42	0.15	1.15–1.75	0.002
Gender (Females *)	0.95	0.30	0.50–1.78	0.858
Ethnicity (Caribbean Black)	0.86	0.19	0.56–1.34	0.500

Outcome; Major Depressive Disorder; * Males as the referent category; OR: Odds Ratio, SE: Standard Error.

**Table 3:** Summary of logistic regression based on ethnicity and gender.

	OR	SE	95% CI	*p*
**African Americans**				
Perception of neighborhood as unsafe	1.24	0.12	1.01–1.53	0.038
Age	1.41	0.16	1.12–1.77	0.005
Gender (Females *)	0.90	0.30	0.45–1.79	0.759
**Caribbean Blacks**				
Perception of neighborhood as unsafe	1.29	0.16	0.98–1.69	0.065
Age	1.57	0.26	1.10–2.24	0.017
Gender (Females *)	1.89	0.86	0.71–5.01	0.183
**Males**				
Perception of neighborhood as unsafe	1.40	0.21	1.03–1.90	0.031
Age	1.54	0.23	1.13–2.10	0.007
Ethnicity (Caribbean Black)	0.48	0.17	0.23–0.99	0.048
**Female**				
Perception of neighborhood as unsafe	1.18	0.12	0.96–1.45	0.112
Age	1.34	0.20	0.99–1.81	0.060
Ethnicity (Caribbean Black)	1.16	0.39	0.59–2.27	0.663

Outcome; Major Depressive Disorder; * Males as the referent category.

**Table 4:** Summary of logistic regression based on the intersection of ethnicity and gender.

	OR	SE	95% CI	*p*
**African American Males**				
Perception of neighborhood as unsafe	1.41	0.22	1.03–1.93	0.034
Age	1.53	0.24	1.11–2.11	0.011
**African American Females**				
Perception of neighborhood as unsafe	1.17	0.12	0.94–1.46	0.144
Age	1.33	0.21	0.96–1.84	0.088
**Caribbean Black Males**				
Perception of neighborhood as unsafe	1.08	0.24	0.67–1.76	0.725
Age	2.00	0.75	0.90–4.45	0.085
**Caribbean Black Females**				
Perception of neighborhood as unsafe	1.34	0.19	0.98–1.83	0.064
Age	1.46	0.32	0.91–2.34	0.110

Outcome; Major Depressive Disorder.
